# Practice facilitation for improving cardiovascular care: secondary evaluation of a stepped wedge cluster randomized controlled trial using population-based administrative data

**DOI:** 10.1186/s13063-016-1547-2

**Published:** 2016-09-05

**Authors:** Catherine Deri Armstrong, Monica Taljaard, William Hogg, Amy E. Mark, Clare Liddy

**Affiliations:** 1 Department of Economics, University of Ottawa, Ottawa, ON Canada; 2 Clinical Epidemiology Program, Ottawa Hospital Research Institute, Ottawa, ON Canada; 3 Department of Epidemiology and Community Medicine, University of Ottawa, Ottawa, ON Canada; 4 C.T. Lamont Primary Health Care Research Centre, Bruyère Research Institute, Ottawa, ON Canada; 5 Department of Family Medicine, University of Ottawa, Ottawa, ON Canada; 6 Institute for Clinical Evaluative Sciences, Ottawa, ON Canada; 7 Ottawa Hospital Research Institute, Ottawa, ON Canada

**Keywords:** Practice facilitation, Primary care, Cardiovascular health

## Abstract

**Background:**

Practice facilitation (PF), a multifaceted approach in which facilitators (external health care professionals) help family physicians to improve their adoption of best practices, has been highly successful. Improved Delivery of Cardiovascular Care (IDOCC) was an innovative PF trial designed to improve evidence-based care for people who have, or are at risk of, cardiovascular disease (CVD). The intervention was found to be ineffective as assessed by a patient-level composite score based on chart reviews from a subsample of patients (*N* = 5292). Here, we used population-based administrative data to examine IDOCC’s effect on CVD-related hospitalizations.

**Methods:**

IDOCC used a pragmatic, stepped wedge cluster randomized controlled design involving primary care providers recruited across Eastern Ontario, Canada. IDOCC’s effect on CVD-related hospitalizations was assessed in the 2 years of active intervention and post-intervention years. Marginal and mixed-effects regression analyses were used to account for the study design and to control for patient, physician, and practice characteristics. Secondary and subgroup analyses investigated robustness.

**Results:**

Our sample included 262,996 patient/year observations representing 54,085 unique patients who had, or were at risk of, CVD, from 70 practices. There was a strong decreasing secular trend in CVD-related hospitalizations but no statistically significant effect of IDOCC. Relative to patients in the control condition, patients in the intervention condition were estimated to have 4 % lower odds of CVD-related hospitalizations (adjOR = 0.96, 99 % CI 0.83 to 1.11). The nonsignificant result persisted across robustness analyses.

**Conclusions:**

Clinical outcomes from administrative databases were examined to form a more complete picture of the (in)effectiveness of a large-scale quality improvement intervention. IDOCC did not have a significant effect on CVD hospitalizations, suggesting that the results from the primary composite adherence score analysis were neither due to choice of outcome nor relatively short follow-up period.

**Trial registration:**

ClinicalTrials.gov NCT00574808, registered on 14 December 2007.

**Electronic supplementary material:**

The online version of this article (doi:10.1186/s13063-016-1547-2) contains supplementary material, which is available to authorized users.

## Background

Practice facilitation (PF) is an approach designed to improve the uptake of evidence-based best practices in primary care [1]. It involves bringing external health care professionals into a practice to help identify areas for improvement, set improvement goals, and provide tools and approaches to reach these goals. Numerous studies have associated PF with improvements in prevention, diabetes care, smoking cessation, and cancer care [2–5]. PF has become popular as evidenced by its broad implementation worldwide [1, 6]. Still, some aspects of PF remain poorly understood [1, 2, 5, 7–9]. More research is needed to explore the impact of PF programs targeting multiple diseases, to examine its effectiveness across different professional settings, and to identify the ideal intensity of intervention (i.e., number of sessions a practice should receive).

The Improved Delivery of Cardiovascular Care (IDOCC) project was an innovative, multifaceted quality improvement trial designed to assist primary care providers in improving their delivery of evidence-based care for patients who have, or are at risk of, cardiovascular disease (CVD) [10]. The 2-year intervention involved sending external outreach facilitators (in this case, specially trained nurses) into participating family medicine practices monthly in the first year and less frequently (every 6–12 weeks) in the second year.

The registered primary outcome of the trial was a composite score assessing physicians’ adherence to recommended care guidelines. This outcome was assessed using chart audit data for approximately 66 randomly selected patients who had, or were at risk of, CVD in each practice. The intervention was found to decrease adherence to evidence-based guidelines (i.e., worsened care) by a statistically significant, but clinically unimportant, amount [11]. These results sharply contrast with findings of previous facilitation trials [5, 12–14].

In that study, annual chart audits ended in the final intervention year – meaning that a slower uptake of best practices could not be captured in the analysis. Here, we used population-based administrative databases to link IDOCC participants to routinely collected clinical outcomes in all study years (pre-intervention, two intervention years, and post-intervention) in order to provide a more complete picture of the potential effect of IDOCC, focusing on CVD-related hospitalizations.

## Methods

### Setting

Facilitation visits took place between 14 April 2008 and 27 March 2012 in the Champlain Local Health Integration Network (LHIN) of Eastern Ontario, Canada, a diverse region of 1.2 million individuals with disease burdens and health outcomes comparable to the rest of the province and country. A complete protocol of the IDOCC study has been published elsewhere [10]. We provide an overview of the methods as per the Consolidated Standards of Reporting Trials (CONSORT) checklist [15] (see Additional file 1).

### Study design

**Fig. 1:**
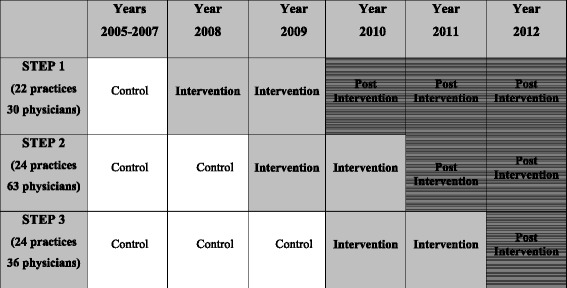
The Improved Delivery of Cardiovascular Care (IDOCC) stepped wedge study design used in the analysis of clinical outcomes using population-based health administrative data. Legend: the darker nonstriped cells indicate IDOCC intervention years and the striped cells indicate post-IDOCC years where patients may still be benefitting from the intervention. Blank cells represent control periods

IDOCC followed a stepped wedge cluster randomized controlled design utilizing three distinct study groups, or “steps.” Practices were allocated to steps by region and each step sequentially started the intervention a year apart (see Fig. 1).

To randomize practices, the LHIN was first divided into nine geographic regions using Geographic Information System mapping technology, and then grouped into three strata (west, central, and east) using computer-generated random numbers provided by an independent statistician. Within each stratum, the regions were randomly assigned to one of three study steps. Thus, each step comprised participating practices from three of the nine regions, one from the east, central, and west.

The stepped wedge design was chosen to (1) minimize the practical, logistical, and financial constraints associated with large-scale project implementation, (2) control for any secular trend in CVD hospitalization rates, and (3) ensure that all practices eventually received the intervention [16]. The trial concluded at the end of the intervention phase for step 3 practices, as scheduled. The Ottawa Hospital Research Ethics Board approved this trial (2007292-01H).

### Recruitment

Invitations to participate were sent to all practices in the LHIN that (1) were in operation for 2 years prior to 2008, and (2) offered general primary care services. Consent was obtained from all participating physicians. Participants received no financial compensation, though could receive continuing professional development credits. All participants agreed to have their data identified within the Institute for Clinical Evaluative Sciences (ICES), the research institute that houses the administrative data. Details on the recruitment strategy have been published elsewhere [10]. Patient consent was not required as the intervention did not directly target patients, and no identifiable information was collected from patients; all analyses were conducted in securely held databases within ICES [17].

### Dataset creation

Datasets were linked using unique encoded patient and physician identifiers and analyzed at ICES. An Appendix containing additional information regarding data linkage and construction as well as all codes from the International Classification of Diseases (ICD) used in identifying patients with CVD and CVD-related hospitalizations is available separately (see Additional file 2).

To construct the sample of patients targeted by IDOCC, we first created a patient roster for each physician for each year (2005 to 2012). Patients officially rostered to a physician for capitation payment reasons were identified using the Client Agency Program Enrollment database. Patients not formally enrolled to a family physician were “virtually” attributed to the family physician who has billed (or “*shadow* billed” in capitation and salaried models) the largest dollar amount of services over a 2-year period. This information is available in the Ontario Health Insurance Plan claims database. This “virtual rostering” approach has been used in other studies and is the accepted reporting approach for Ontario’s Ministry of Health and Long-Term Care reports [18, 19]. Physician characteristics (sex, years since graduation, and indicator for being trained abroad) and practice characteristics (remuneration model type, rurality indicator, and practice group size) were obtained from the ICES Physician Database.

Next, we identified all patients within each roster who had, or were at risk of, CVD. Using validated algorithms, we identified patients aged 40 years and over who had coronary artery disease, cerebral vascular disease (to capture transient ischemic attack and stroke), diabetes, renal failure, or peripheral vascular disease. Patients at risk of CVD were identified as men aged 45 or older or women aged 55 years or older with hypertension. Patient characteristics (age, sex, number of Aggregated Diagnosis Groups (ADGs), rurality, income quintile and immigrant status) were obtained from the Registered Persons Database and Postal Code Conversion File. Johns Hopkins ADGs measure patients’ comorbidity. Based on their health care utilization in the previous 2 years, patients are attributed between zero and 32 ADGs, with greater number of ADGs indicating greater comorbidity. Each ICD code is assigned to one of the 32 ADGs based on five clinical dimensions: duration, severity and etiology of the condition, diagnostic certainty, and specialty care involvement [20].

### Outcome measure

The clinical outcome of interest was a patient-level dichotomous indicator of any hospital admission for CVD in the fiscal year (April–March) and was constructed from information in the Discharge Abstract Database. The reason for hospitalization was identified using the ICD10-CA codes listed under the hospitalization’s “Most Responsible Diagnosis” (see Additional file 2 for the list of ICD codes used to identify the various CVDs). The outcome was assessed cross-sectionally using all eligible patients in each study year (2005–2012).

### Sample size

The sample size for the IDOCC study was determined based on the primary process measure for the trial (adherence) as presented elsewhere [11]. For this secondary analysis, the clinical outcome was assessed in all participating practices using population-level data, and no additional a-priori sample size calculation was carried out.

### Data analysis

The number and percentage of patients with each of the five cardiovascular diseases and who were considered “at risk” is reported by study year. “Baseline” descriptive statistics summarizing practice and patient characteristics in 2007, the year before the first set of practices received the intervention, are reported by step. CVD hospitalizations were analyzed at the individual patient level using generalized linear mixed effects regression with logit link function and binomial distribution, estimated using residual pseudo-likelihood in SAS v.9.3. Region (unit of randomization), time, and treatment were specified as fixed effects, while practices and providers were specified as random effects to account for multiple levels of clustering of patients within providers and practices. Treatment was defined as a three-level categorical variable to allow for control, intervention (2 years of active intervention), and post-intervention conditions, while time was modelled as a simple linear term after visual inspection of empirical logit plots of the observed trends in hospitalization. The statistical significance of the intervention was assessed using approximated Wald F-tests with denominator degrees of freedom estimated using the between-within method [21]. Pairwise differences among the three intervention conditions were calculated, expressed as odds ratios (OR) with 99 % confidence intervals (CIs). The unadjusted analysis was followed by two adjusted analyses: the first controlling for patient characteristics and the second controlling for patient, physician, and practice characteristics.

Three additional analyses were performed to gauge robustness of the results. First, CVD hospitalizations were measured as an annual count as opposed to a dichotomous outcome and analyzed using negative binomial distribution and log link function. Due to failure of the mixed-effects model to converge, a marginal model using generalized estimating equations (GEE) was used with robust standard errors and an exchangeable correlation structure to account for clustering of patients within practices. Results from the marginal model are presented using relative risk (RR) with 99 % CI for pairwise comparisons.

The second robustness specification restricted the sample to patients with diabetes. This subgroup was chosen for three reasons: (1) patients with diabetes in the health utilization datasets can be identified very accurately, decreasing possible measurement error, (2) patients with diabetes comprise the largest diagnosis group in our sample, and (3) the majority of practices were working with the facilitators on diabetes-related issues. The third robustness specification included only individuals from the sample who had diagnosed CVD; individuals identified as “at risk” were excluded to assess sensitivity to potential misclassification of this group due to the known limitations of health administrative data. The model for the second and third robustness analyses were as described for the main model.

## Results

Of the 434 eligible practices, 93 (comprising 194 physicians) agreed to participate in IDOCC. Ten practices withdrew from the study prior to the initiation of the intervention. Twelve of the participating practices were community health centers and were excluded from this analysis, as reliable information on this practice model is only available within ICES from 2008 onward. Two practices were long-term care facilities served by the same family physician and, thus, collapsed into a single location for practical purposes. Other challenges we faced in creating linkages included an inability to create a virtual roster for physicians who took eight or more consecutive weeks away from work and a lack of billing data. After accounting for these exclusions, we were able to successfully link to the outcomes of patients in 70 practices (comprising 129 physicians). The total number of patients linked each year for analysis ranged from 26,042 to 37,050, with almost half (47.8 to 51.5 %) being identified as at risk of CVD (Table 1).

**Table 1:** Number and percentage of eligible patients at risk of, and with, cardiovascular disease (CVD) by year and type of chronic condition

	2005	2006	2007	2008	2009	2010	2011	2012
Total number of patients	26,042	28,196	30,308	32,901	34,898	36,610	37,050	36,991
At risk of CVD	13,424	14,500	15,241	16,312	17,211	17,817	17,797	17,671
	(51.5 %)	(51.4 %)	(50.3 %)	(49.6 %)	(49.3 %)	(48.7 %)	(48.0 %)	(47.8 %)
Patients with CVD^a^								
Coronary artery disease	4533	4871	5231	5733	6084	6440	6607	6634
	(17.4 %)	(17.3 %)	(17.3 %)	(17.4 %)	(17.4 %)	(17.6 %)	(17.8 %)	(17.9 %)
Cerebral vascular disease	980	1045	1090	1161	1155	1147	1125	1088
	(3.8 %)	(3.7 %)	(3.6 %)	(3.5 %)	(3.3 %)	(3.1 %)	(3.0 %)	(2.9 %)
Diabetes	8143	8927	9976	11063	11906	12784	13223	13411
	(31.3 %)	(31.7 %)	(32.9 %)	(33.6 %)	(34.1 %)	(34.9 %)	(35.7 %)	(36.3 %)
Renal failure	1499	1737	1970	2331	2426	2587	2578	2511
	(5.8 %)	(6.2 %)	(6.5 %)	(7.1 %)	(7.0 %)	(7.1 %)	(7.0 %)	(6.8 %)
Peripheral vascular disease	249	256	269	288	317	330	332	325
	(1.0 %)	(0.9 %)	(0.9 %)	(0.9 %)	(0.9 %)	(0.9 %)	(0.9 %)	(0.9 %)
Hypertension	21,730	23,557	25,356	27,486	29,110	30,537	30,874	30,812
	(83.4 %)	(83.5 %)	(83.7 %)	(83.5 %)	(83.4 %)	(83.4 %)	(83.3 %)	(83.3 %)

^a^Percentages do not add up to 100 because patients can have more than one condition

The baseline descriptive data are presented in Table 2. While similar numbers of practices were included in each step (22 in step 1 and 24 in steps 2 and 3), there were substantial differences across steps with respect to practice and patient characteristics. Step 1 practices had the lowest prevalence of female doctors (40 % versus 64 % in step 2) and the highest prevalence of being located in a rural area (37 % versus <5 % in step 2), and were almost exclusively fee-for-service (as compared to approximately 50 % in steps 2 and 3). Patients from step 1 practices were most commonly rural (39 % versus 5 % for step 3) and had the highest number of annual hospital admissions for CVD in 2007.

**Table 2:** Comparison of practice, provider, and patient characteristics at baseline (2007) by Step

Characteristic	Step 1	Step 2	Step 3
Practice			
Number of practices (*n*)	22	24	24
Provider			
Number of providers (*n*)	30	63	36
Female (*n*, %)	12 (40.0 %)	40 (63.5 %)	21 (58.3 %)
Years since graduation from 2007 (mean, SD)	25.5 (9.9)	18.9 (9.2)	24.1 (10.1)
Primary care model (*n*)			
Fee for service	25–29	26	19
Capitation, non-FHT	1–5	23	10–14
Capitation, FHT	0	14	1–5
Rural practices (*n*, %)	11 (36.7 %)	1–5 (1.0–5.0 %)	11 (30.6)
Patient			
Number of patients (*n*)	7830	12,819	9659
Age (mean, SD)	67.9 (12.7)	65.0 (11.9)	66.9 (12.0)
Female (*n*, %)	4069 (52.0 %)	6669 (52.0 %)	5074 (52.5 %)
Number of Aggregated Diagnostic Groups (mean, SD)	7.1 (3.7)	6.8 (3.5)	6.7 (3.6)
Rural residents (n, %)	3071 (39.2 %)	618 (4.8 %)	2558 (26.5 %)

*FHT* Family Health Team

**Fig. 2:**
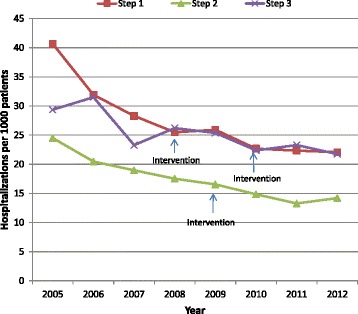
Observed cardiovascular disease (CVD) hospitalization rates among all patients with, or at risk of, CVD

The observed trends in CVD hospitalization rates are presented in Fig. 2. There were differences in the levels of hospitalizations across steps, but a similar decreasing secular trend. The results from the mixed-effects logistic regression analysis with and without adjustment for covariates are presented in Table 3. The analysis included 262,996 observations representing 54,085 unique patients. The effect of the intervention was not statistically significant (*p* = 0.78 in the unadjusted and *p* = 0.67 in the fully adjusted model). Pairwise least square mean differences in hospitalization proportions between intervention conditions are presented in Table 4 panel a. Estimates obtained from analyses adjusting for patient, provider, and practice characteristics were similar to those from unadjusted analyses. On average, patients in the intervention condition had a 4 % lower odds of any CVD hospitalizations relative to the control condition (adjOR = 0.96, 99 % CI 0.83 to 1.11), and a 7 % lower odds in the post-intervention condition relative to control (adjOR = 0.93, 99 % CI 0.74 to 1.15), though neither difference was statistically significant (*p* = 0.49 and *p* = 0.36 respectively).

**Table 3:** Primary outcome analysis of any cardiovascular disease (CVD) hospitalization using mixed-effects logistic regression analysis accounting for clustering by practice and provider (*N* = 262,996)

Parameter	Model A		Model B		Model C	
	(Unadjusted)		(Adjusted for patient factors)		(Adjusted for patient and provider factors)	
	Odds ratio	*p* value	Odds ratio	*p* value	Odds ratio	*p* value
Phase						
Baseline	Ref		Ref		Ref	
Intervention	0.96	0.51	0.96	0.50	0.96	0.49
Post	0.95	0.51	0.93	0.37	0.93	0.36
Year	0.95	<0.001	0.95	0.001	0.95	0.002
Region						
1	1.54	0.04	1.13	0.49	0.87	0.41
2	0.89	0.48	0.90	0.43	0.75	0.028
3	1.08	0.65	0.99	0.94	0.81	0.09
4	1.36	0.07	1.01	0.93	0.74	0.035
5	0.84	0.34	0.90	0.50	0.85	0.22
6	0.87	0.51	0.95	0.75	0.89	0.47
7	0.80	0.18	0.81	0.11	0.72	0.011
8	0.95	0.81	0.92	0.65	0.83	0.24
9	Ref		Ref		Ref	
Patient characteristics						
Age			1.03	<0.001	1.03	<0.001
Sex			0.55	<0.001	0.55	<0.001
Number of ADGs						
0			Ref	-	Ref	-
1 (1–4)			0.78	0.18	0.78	0.19
2 (5–9)			1.16	0.44	1.16	0.42
3 (10+)			2.03	<0.001	2.04	<0.001
Location						
Urban			Ref	-	Ref	-
Suburban			1.21	0.001	1.08	0.24
Rural			1.23	0.001	1.12	0.13
Income quintile						
1			Ref	-	Ref	-
2			0.89	0.015	0.90	0.020
3			0.86	0.002	0.86	0.002
4			0.80	<0.001	0.80	<0.001
5			0.75	<0.001	0.75	<0.001
Immigrant			1.00	0.995	0.997	0.97
Provider characteristics						
Payment model						
FFS					Ref	-
Capitation, FHT					0.97	0.68
Capitation, non-FHT					1.07	0.13
Female physician					0.90	0.058
Years since graduation					1.00	0.24
Physician rurality						
Urban					Ref	-
Suburban					1.46	0.013
Rural					1.33	0.032
Trained abroad					1.21	0.041
Practice size					1.02	0.054

*ADG* Aggregated Diagnostic Groups, *FHT* Family Health Team

**Table 4:** Pairwise least square mean comparisons between intervention conditions from primary outcome analysis and three robustness analyses

	Unadjusted			Adjusted for patient characteristics			Adjusted for patient and physician		
	OR	99 % CI	*p* value	OR	99 % CI	*P* value	OR	99 % CI	*P* value
**a) Primary outcome analysis: any CVD hospitalization (Yes/No)** **(** ***N*** ** = 262,996)** ^**1**^									
Post vs. intervention	0.98	0.86–1.12	0.73	0.96	0.85 − 1.10	0.48	0.96	0.84 − 1.10	0.46
Intervention vs. baseline	0.96	0.83 − 1.12	0.51	0.96	0.83 − 1.12	0.50	0.96	0.83 − 1.11	0.49
Post vs. baseline	0.95	0.76 − 1.18	0.51	0.93	0.75 − 1.15	0.37	0.93	0.74 − 1.15	0.36
	Unadjusted			Adjusted for patient characteristics			Adjusted for patient and physician		
	RR	99 % CI	*p* value	RR	99 % CI	*p* value	RR	99 % CI	*p* value
**b) Number of CVD hospitalizations per year (** ***N*** ** = 262,996)** ^**2**^									
Post vs. intervention	0.99	0.84 − 1.19	0.99	0.97	0.82 − 1.15	0.72	0.97	0.83 − 1.15	0.76
Intervention vs. baseline	0.96	0.83 − 1.12	0.61	0.95	0.83 − 1.19	0.50	0.95	0.83 − 1.09	0.46
Post vs. baseline	0.96	0.72 − 1.28	0.78	0.92	0.71 − 1.21	0.57	0.93	0.71 − 1.21	0.56
	Unadjusted			Adjusted for patient characteristics			Adjusted for patient and physician		
	OR	99 % CI	*p* value	OR	99 % CI	*p* value	OR	99 % CI	*P* value
**c) Primary outcome analysis of diabetic patient subsample (** ***N*** ** = 89,433)** ^**3**^									
Intervention vs. baseline	1.09	0.88 − 1.36	0.29	1.09	0.87 − 1.36	0.33	0.97	0.79 − 1.18	0.70
Post vs. baseline	1.11	0.80 − 1.54	0.40	1.07	0.77 − 1.49	0.58	1.09	0.87 − .36	0.34
Post vs. intervention	1.02	0.83 − 1.24	0.84	0.99	0.81 − 1.20	0.86	1.05	0.76 − 1.46	0.68
	Unadjusted			Adjusted for patient characteristics			Adjusted for patient and physician		
	OR	99 % CI	*p* value	OR	99 % CI	*p* value	OR	99 % CI	*P* value
**d) Primary outcome analysis excluding patients only at risk of CVD (** ***N*** ** = 132,686)** ^**4**^									
Intervention vs. baseline	1.04	0.89 − 1.21	0.56	1.01	0.87 − 1.18	0.85	1.00	0.86 − 1.17	0.98
Post vs. baseline	1.06	0.89 − 1.25	0.42	1.05	0.89 − 1.25	0.44	1.05	0.88 − 1.25	0.44
Post vs. intervention	1.09	0.85 − 1.41	0.37	1.07	0.83 − 1.38	0.52	1.05	0.82 − 1.36	0.59

*CI* confidence interval. *CVD* cardiovascular disease. *OR* odds ratio. *RR* relative risk

^1^Mixed-effects logistic regression analysis, accounting for clustering by practice and provider

^2^Negative binomial regression analysis using robust standard errors accounting for clustering by practice

^3^Mixed-effects logistic regression analysis, accounting for clustering by practice and provider

^4^Mixed-effects logistic regression analysis, accounting for clustering by practice and provider

Tables 4 panels b, c and d present the results from our robustness analyses. Modeling the outcome as a count (Table 4 panel b) indicated statistically insignificant estimates of effectiveness: a 5 % reduction in adjusted risk of one additional CVD hospitalization in an intervention year versus baseline (RR = 0.95, 99 % CI 0.83 to 1.09, *p* = 0.46) and a 7 % reduction in a post-intervention year versus baseline (RR = 0.93, 99 % CI 0.71 to 1.21, *p* = 0.56). Both approaches led to the same conclusion: no statistically significant effect of IDOCC on CVD hospitalizations. The final two robustness checks restrict the sample to patients with diabetes (Table 4 panel c) and then to patients with diagnosed CVD (excluding those only at risk) (Table 4 panel d). In no case was IDOCC found to have had any meaningful effect on CVD hospitalizations.

## Discussion

Our analysis addressed several limitations of the original IDOCC study [11]: we were able to (1) analyze population-level data as opposed to a smaller sample of chart audit data, (2) examine outcomes in a larger number of time periods including post-intervention years to allow for the possibility of slower uptake in best practices, and (3) focus on CVD hospitalizations, a clinical outcome which may be more relevant than the composite score.

We found a strong decreasing secular trend in CVD hospitalizations, but no significant effect of the intervention on hospitalizations in either intervention or post-intervention years. These results cohere with the previously published analysis, which used chart audit data to evaluate the program’s impact on providers’ adherence to recommended care guidelines as measured by a composite score of eight indicators. The intervention was found to decrease adherence to evidence-based guidelines by a statistically significant, but clinically insignificant, amount [11].

Other reasons for the lack of effect remain to be explored. One explanation is that the intended number of face-to-face facilitator visits was not achieved. While 13 visits were planned for the first intervention year, on average only 6.6 visits per year were made. The reasons for this shortfall include: competing clinical priorities, practice disruptions (i.e., system upgrades), a reduction in the number of facilitators due to budget reductions and the H1N1 outbreak which may have diverted resources.

A second explanation might be the “rising tide” phenomenon whereby the same pressures that trigger the development of a quality improvement intervention also drive spontaneous, system-wide changes that lead to across-the-board improvements [22]. In such circumstances, controlled evaluations may detect no incremental benefit of the intervention. This explanation is plausible given the clearly decreasing secular trend in hospitalizations.

A third explanation may be the broad focus of the intervention. In contrast to targeting a single disease, IDOCC targeted guidelines for patients with a broad number of cardiovascular-related conditions and risk factors. This broad focus may have impeded practices’ ability to implement focused, system-level changes, or may have diverted resources away from one area at the expense of another. We are exploring these issues with an in-depth qualitative study of participating practices.

A fourth explanation may be that we lacked study power. We conducted no a-priori power calculations for the secondary clinical outcomes, but given 70 participating practices (22 in step 1, and 24 in each of steps 2 and 3), an average of 180 patients per practice per year over 8 years, and an intracluster correlation coefficient of 0.001 (or coefficient of variation of 0.2), application of the power formulas described by Hussey and Hughes (2007) [23] showed that we had 82 % power to detect an OR of 0.76, i.e., a reduction in CVD hospitalizations from a baseline proportion of 25 per 1000 patients to 19 per 1000 patients. Since these power formulas assumed an underlying model with time specified as a categorical variable, we may have been able to detect a slightly smaller difference using our model with time as a simple linear term.

Our study had several strengths, including implementation across a large geographic area involving a diverse range of practices, with outcomes assessed using administrative data for a substantial patient population. The stepped wedge design allowed us to maintain a robust randomized controlled trial model while offering the intervention to all practices.

Our study also had some limitations. First, we had to exclude from our analysis the 12 community health centers that underwent the IDOCC intervention as reliable administrative information on this practice model was not available for the entire study period. Second, we were unable to identify patients at risk of CVD using the same criteria as in the chart audit study, which included smoking status and dyslipidemia, characteristics not easily captured in administrative data, as risk factors. Restricting the sample to those with confirmed CVD and then further to those with diabetes (the most common and best identified disease with existing algorithms), did not change the results. Third, we encountered several computational challenges due to the sheer size of the datasets and the complexity of the models. As a result, we were unable to account for the correlation in repeated measures on the same patients over time which decreased our ability to detect any potential effect of the intervention.

Fourth, due to practical and logistical constraints we were unable to randomize individual practices. Randomizing at a regional level led to some imbalances in the characteristics of participants across steps. To the extent that our adjusted analyses failed to capture these underlying differences, our estimates may be biased. Fifth, we did not directly address the possibility of death in our analysis. Recall that our outcome measure included any patient who was hospitalized for CVD in each study year, even if they subsequently died. Patients who died without prior CVD hospitalizations would have contributed to the denominators for that year, but not the numerators. To the extent that such patients are a biased subsample of all patients with identical covariate values who are at risk of hospitalization in any given year, our parameter estimates may be biased (i.e., residual informative censoring).

## Conclusion

IDOCC did not appear to significantly impact CVD hospitalizations. Our findings form a more complete picture of the (in)effectiveness of IDOCC and clarify that the null result reported in the earlier paper was neither due to choice of composite score outcome nor the relatively short follow-up period [11]. PF is an expensive and resource-intensive way to facilitate change in physician behaviour, but—if effective—can create overall health system savings [24]. As the popularity and expectation of PF continues to grow, results from trials such as this are crucial to understanding the scenarios under which PF can be considered an efficient use of scare health care resources.

## Additional files

Additional file 1: CONSORT Extension for Cluster Trials Checklist. (PDF 341 kb) Additional file 2: Additional documentation regarding Dataset construction. (PDF 313 kb)

## Abbreviations

CVD: Cardiovascular disease
ICES: Institute for Clinical Evaluative Sciences
IDOCC: Improved Delivery of Cardiovascular Care
LHIN: Local Health Integration Network
PF: Practice facilitation.
