# Supplementary material for: Practice facilitation for improving cardiovascular care: secondary evaluation of a stepped wedge cluster randomized controlled trial using population-based administrative data
Source: Trials. 2016 Sep 5;17(1):434. doi: 10.1186/s13063-016-1547-2 (PMC5011906; doi:10.1186/s13063-016-1547-2)
Supplement: Additional file 2: — Additional documentation regarding Dataset construction. (PDF 313 kb) [file 13063_2016_1547_MOESM2_ESM.pdf]

Data Construction Appendix

Data Sources: All data sources upon which this project relies are regularly collected administrative health encounter data housed at the Institute for Clinical Evaluative Sciences (ICES). The following data set are used in this study.

1) **CAPE**: The Client Agency Program Enrolment database tracks patient enrolment to individual family physicians.
2) **DAD**: The Discharge Administrative Database supplies information on acute care hospitalizations
3) **IPDB**: ICES Physician Database contains annual demographic data for physicians as well as information on areas of specialization and workload.
4) **NACRS**: The National Ambulatory Care Reporting System provides information on all emergency room encounters.
5) **OHIP**: The Ontario Health Insurance Program billing claims system captures provision of care to patients.
6) **PCCF+**: Statistics Canada's Postal Code Conversion File allows patients to be attributed to a census dissemination area based on postal code.
7) **RPDB**: The Registered Person's Database captures patient demographic information, including age, sex and postal code.

Data Linkage – Graphical Summary:

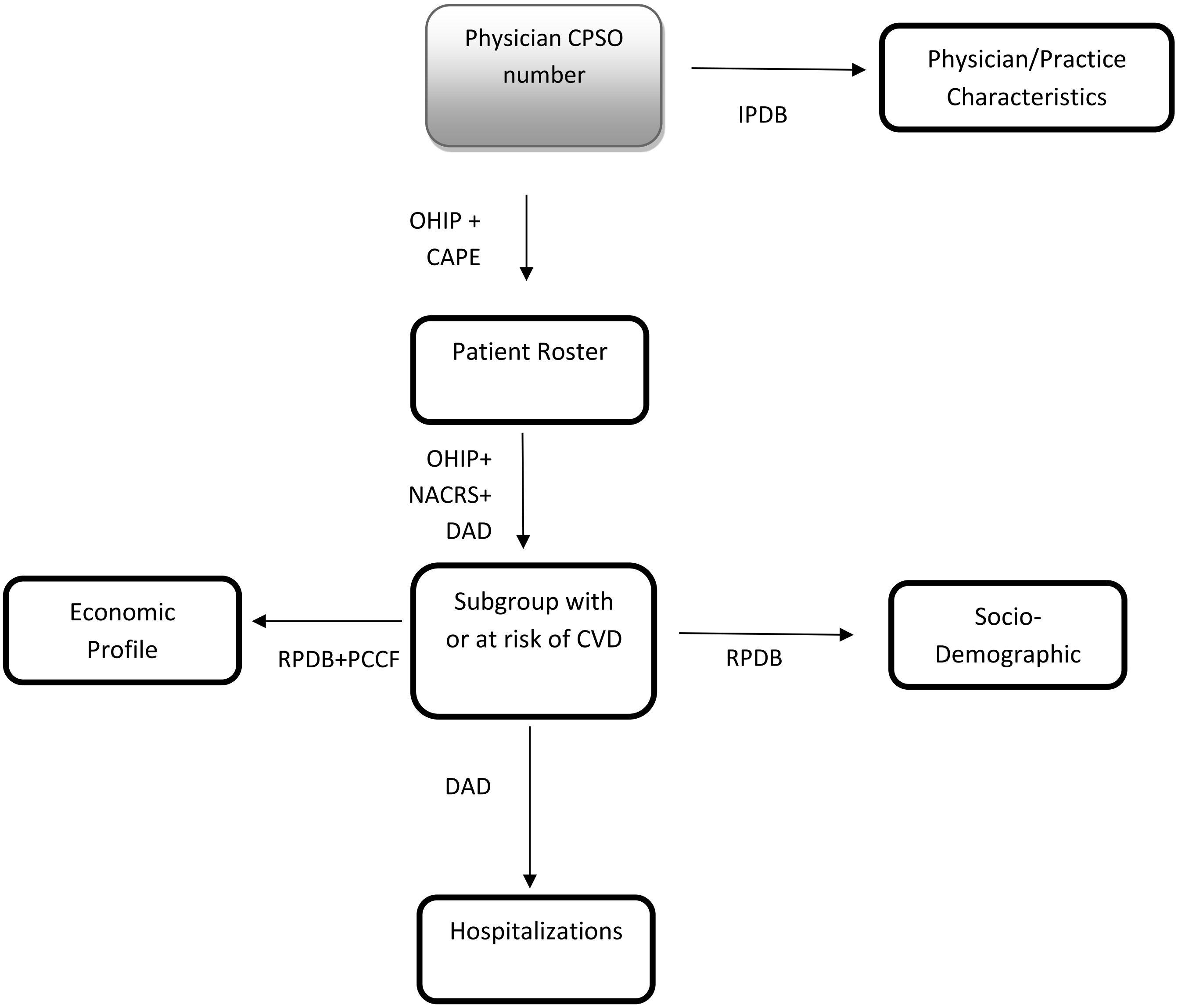

Summary of Algorithms used to identify patients with CVD

Coronary Artery Disease (CAD)	
**Data Sources**	DAD/SDS (April 1988 forward)<br>NACRS (SDS only) (F2003-04 forward)<br>OHIP (July 1991 forward)
**Case Ascertainment**	• 1 hospital admission with a CAD diagnosis code, or 1 CAD OHIP billing code
**Codes Used**	Acute myocardial infarction[1]<br>ICD-9: 410<br>ICD-10: I21, I22, I252, I513<br><br>Percutaneous coronary invervention[2]<br>prcode: 4802, 4803, 4809<br>incode: 1IJ50, 1IJ57GQ, 1IJ57GS<br><br>Coronary artery bypass graft surgery[2]<br>prcode: 481<br>incode: 1IJ76<br><br>Cardiac cathetrerization<br>prcode: 4892, 4893, 4894, 4895, 4896, 4897, 4898, 4995, 4996, 4997<br>incode: 3IP10<br><br>OHIP billing codes: Z442 or G297
**Notes**	Definition from Tu et al.[3]

Cerebral Vascular Disease (CVD)[4]	
**Data Sources**	DAD/SDS (April 1988 forward)<br>NACRS (SDS only) (F2003-04 forward)
**Case Ascertainment**	• 1 hospital admission with a CVD diagnosis code
**Codes Used**	ICD-9: 430, 431, 432, 434, 436 (any type)<br>ICD-10-CA: I63, I64, G46, G45, H34 (any type)

Ontario Diabetes Database (ICES Derived Cohort)[5,6]	
**Case Ascertainment (Bridge)**	• 1 or 2 OHIP dxcode claims prior to 19th birth date **AND**<br>• 1 OHIP claim after 19th birth date within 2 years<br>*(19th birth date is used as incident date)*
**Case Ascertainment (Adult)**	• 2 OHIP dxcodes **OR**<br>• 1 OHIP feecode **OR**<br>• 1 CIHI admission after 19th birth date

**Data Sources**	OHIP (July 1991 forward)<br>DAD/SDS (April 1988 forward)<br>NACRS (SDS only) (F2003-04 forward)
**Codes Used**	ICD-9: 250 (any type)<br>ICD-10-CA: E10, E11, E13, E14 (any type)<br>OHIP dxcode: 250<br>OHIP feecode: Q040, K029, K030
**Notes**	Gestational Diabetes records excluded<br>Incidence not reported for first 3 years

**Renal Failure**	
**Case Ascertainment**	• Any one of the 2 OHIP diagnosis codes or 9 ICD-9/ICD-10 codes within the 5 years prior to cohort entry without kidney transplant within the previous five years and without dialysis within the previous 1 year.
**Data Sources**	DAD/SDS (April 1988 forward)<br>NACRS (SDS only) (F2003-04 forward)<br>OHIP (July 1991 forward)
**Inclusion Codes Used**	ICD-9: 4030, 4031, 4039, 4040, 4041, 4049, 585, 586, 5888, 5889, 2504 (any dxcode)<br>ICD-10-CA: E102, E112, E132, E142, I12, I13, N08, N18, N19<br>OHIP: 403, 585
**Exclusion Codes Used**	**Kidney Transplant:**<br>ICD-9: V420, 99681<br>CCP: 6743, 675<br>ICD-10: T861, N165, Z940<br>CCI: 1PC85<br>OHIP: E762, E769, E771, G347, G348, G408, G409, G412, S434, S435, Z631<br><br>**Dialysis Codes:**<br>ICD-9: E1022, E1023, E1122, E1123, E1322, E1323, E1422, E1423, N180, T824, Y602, Y612, Y622, Y841, Z49, Z992<br>CCP: 5127, 5142, 5143, 5195, 6698<br>ICD-10: V451, V560, V568, 36104<br>CCI: 1PZ21, 1OT53DATS, 1OT53HATS, 1OT53LATS, 1SY55LAFT, 7SC59QD, 1KY76<br>OHIP: G082, G083, G085, G090, G091, G092, G093, G094, G095, G096, G099, G294, G295, G323, G324, G325, G326, G327, G330, G331, G332, G333, G336, G860, G861, G862, G863, G864, G865, G866, H540, H740, R825, R826, R827, R833, R840, R841, R843, R848, R849, R850, R851, R852, R853, R854, R885, Z450, Z451,Z452
**Notes**	From Fleet JL et al[7]

**Peripheral Vascular Disease (PVD)[4]**

**Case Ascertainment**	• 1 hospital admission with a PVD intervention code **AND**<br>• Without specified diagnosis codes on the same abstract
**Data Sources**	DAD/SDS (April 1988 forward)<br>NACRS (SDS only) (F2003-04 forward)
**Codes Used**	*Major:*<br>• CCP: 96.14, 96.15<br>• CCI: 1VQ93, 1VC93, 1VG93<br>*Minor:*<br>• CCP: 96.11, 96.12, 96.13 **WITHOUT**<br>o ICD-9: 170, 171, 213, 730, 740-759, 800-900, 901-904, 940-950 **on the abstract**<br><br>• CCI: 1WL93, 1WA93, 1WE93, 1WJ93, 1WM93 **WITHOUT**<br>o ICD-10-CA: C40, C41, C46.1, C47, C49, D160, M46.2, M86, M87, M89.6, M90.0-M90.5, Q00, Q38-Q40, S02.0, S04.0 S09.0, S15, S25, T26 **on the abstract**<br>*Bypass:*<br>• CCP: 51.25, 51.29, 50.18 ICD9 **WITHOUT**<br>o ICD-9: 4141, 441, 442 **on the abstract**<br>• CCI: 1KG50, 1KG57, 1KG76, 1KG35HAC1, 1KG35HHC1 **WITHOUT**<br>o ICD-10-CA: I67.1, I71, I72, I60, I77.0, I79.0, Q codes **on the abstract**

**Hypertension (ICES Derived Cohort)[8]**	
**Case Ascertainment**	• 1 hospital admission with a hypertension diagnosis code **OR**<br>• 1 OHIP record with a hypertension diagnosis code, followed within 2 years by another OHIP record or a hospital admission with a hypertension diagnosis code
**Data Sources**	OHIP (July 1991 forward)<br>DAD/SDS (April 1988 forward)<br>NACRS (SDS only) (F2003-04 forward)
**Codes Used**	ICD-9: 401x, 402x, 403x, 404x, 405x (any type)<br>ICD-10-CA: I10, I11, I12, I13, I15 (any type)<br>OHIP dxcode: 401, 402, 403, 404, 405 (any type)
**Notes**	Gestational hypertension records excluded<br>Generic exclusions do not apply to this cohort

References

1) Austin PC1, Daly PA, Tu JV. A multicenter study of the coding accuracy of hospital discharge administrative data for patients admitted to cardiac care units in Ontario. Am Heart J 2002;144(2):290-6.
2) Lee DS, Stitt A, Wang X, Yu JS, Gurevich Y, Kingsbury KJ, Austin PC, Tu JV. Administrative hospitalization database validation of cardiac procedure codes. Med Care 2013;51(4):e22-6.

3) Tu JV, Chu A, Donovan LR, Ko DT, Booth GL, Tu K, et al. The Cardiovascular Health in Ambulatory Care Research Team (CANHEART): using big data to measure and improve cardiovascular health and healthcare services. Circ Cardiovasc Qual Outcomes 2015;8(2):204-12.
4) Iron K, Gerson A, Lu H, Manuel D. Using Linked Administrative Data to Assess the Clinical and System Impact of Chronic Disease in Ontario. Healthc Q 2011;14(3):23-27.
5) Hux J, Ivis F, Flintoft V, Bica A. Diabetes in Ontario: Determination of prevalence and incidence using a validated administrative data algorithm. Diabetes Care 2002;25:512-6.
6) Guttman A, Nakhla M, Henderson M, To T, Daneman D, Cauch-Dudek K, et al. Validation of a health administrative data algorithm for assessing the epidemiology of diabetes in Canadian children. Pediatric Diabetes 2010 Mar;11(2):122-8.
7) Fleet JL1, Dixon SN, Shariff SZ, Quinn RR, Nash DM, Harel Z, Garg AX. Detecting chronic kidney disease in population-based administrative databases using an algorithm of hospital encounter and physician claim codes. BMC Nephrol 2013;14:81.
8) Tu K, Campbell NR, Chen Z, Cauch-Dudek K, McAlister FA. Accuracy of administrative databases in identifying patients with hypertension. Open Med 2007;1(1):18-26.
